# Supplementary material for: Shared Neurodevelopmental Perturbations Can Lead to Intellectual Disability in Individuals with Distinct Rare Chromosome Duplications
Source: Genes (Basel). 2021 Apr 23;12(5):632. doi: 10.3390/genes12050632 (PMC8146713; doi:10.3390/genes12050632)

S1 Chromosomal Microarray Analysis ratio profiles samples from the three patients with duplicate genomic region (dup 8q24.13q24.3; dup 18p11.32p11.21 and dupXq22.3-q27).

(Chr8:126,397,316-143,577,971)

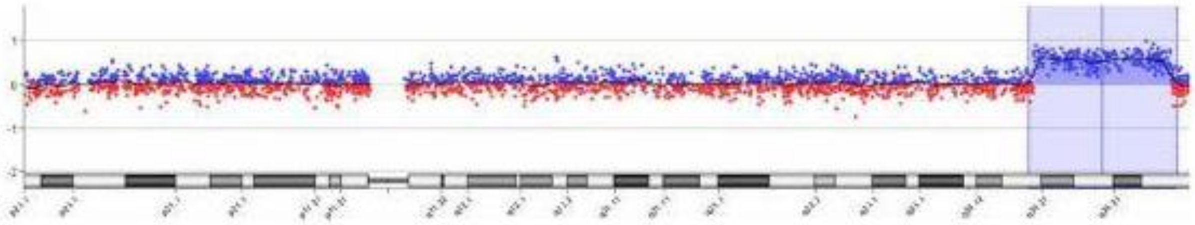

(Chr18:14,316-14,773,575)

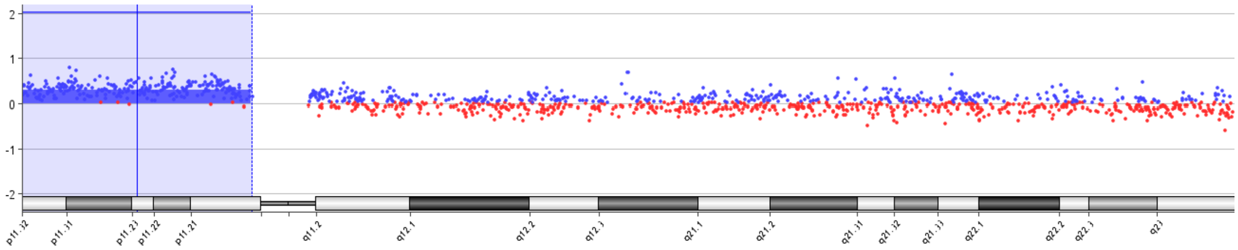

(Chrx:106,283,188-140,340,737)

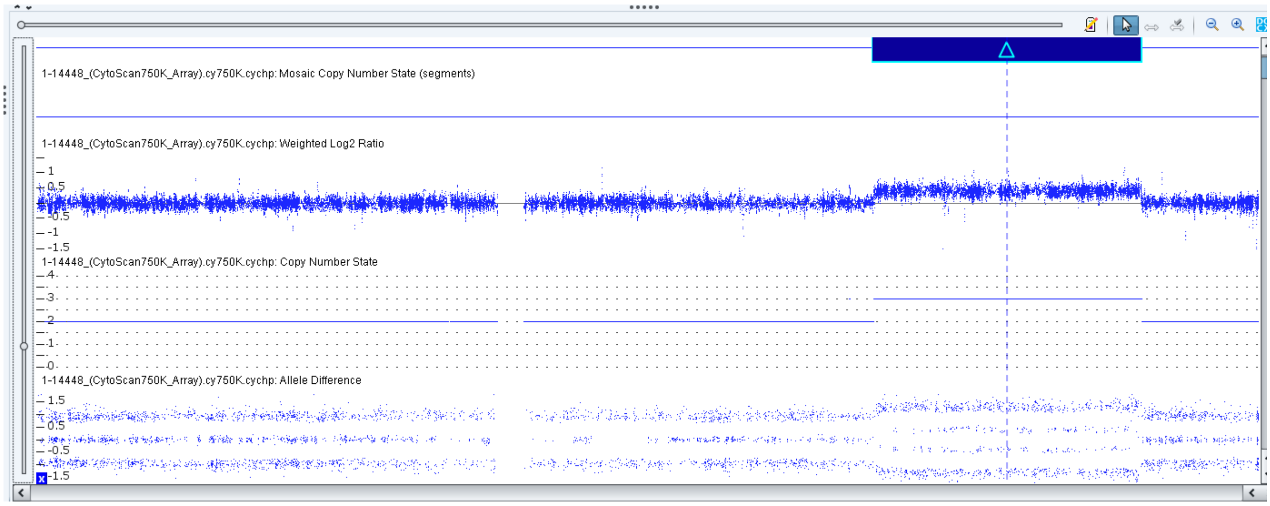

Supplement: Supplementary file 1 [file genes-12-00632-s001.zip › Supplementary material/S1 rev MR.pdf]
